# Optimization of the Codon Pair Usage of Human Respiratory Syncytial Virus Paradoxically Resulted in Reduced Viral Replication *In Vivo* and Reduced Immunogenicity

**DOI:** 10.1128/JVI.01296-19

**Published:** 2020-01-06

**Authors:** Cyril Le Nouën, Cindy L. Luongo, Lijuan Yang, Steffen Mueller, Eckard Wimmer, Joshua M. DiNapoli, Peter L. Collins, Ursula J. Buchholz

**Affiliations:** a Laboratory of Infectious Diseases, National Institute of Allergy and Infectious Diseases, National Institutes of Health, Bethesda, Maryland, USA; b Codagenix Inc., Stony Brook, New York, USA; c Department of Molecular Genetics and Microbiology, Stony Brook University, Stony Brook, New York, USA; St. Jude Children's Research Hospital

**Keywords:** codon pair optimization, genome recoding, negative-strand RNA virus, respiratory syncytial virus

## Abstract

We subjected various open reading frames (ORFs) in the genome of respiratory syncytial virus (RSV) to codon pair optimization (CPO) by increasing the content of codon pairs that are overrepresented in the human genome without changing overall codon usage and amino acid sequences. CPO has the potential to increase the expression of the encoded protein(s). Four viruses were made: Max A (with CPO of NS1, NS2, N, P, M, and SH ORFs), Max B (with CPO of G and F), Max L (with CPO of L), and Max FLC (with CPO of all ORFs except M2-1 and M2-2). Because of the possibility of increased viral replication, each CPO virus was attenuated by the inclusion of a codon deletion mutation (Δ1313) and a missense mutation (I1314L) in the L polymerase. CPO had no effect on multicycle virus replication *in vitro*, temperature sensitivity, or specific infectivity. Max A and L, which in common had CPO of one or more ORFs of proteins of the polymerase complex, exhibited global increases in viral protein synthesis. Max B alone exhibited decreased protein synthesis, and it alone had reduced single-cycle virus replication *in vitro*. All CPO RSVs exhibited marginal reductions in replication in mice and hamsters. Surprisingly, the CPO RSVs induced lower levels of serum RSV-neutralizing antibodies in hamsters. This reduced immunogenicity might reflect reduced viral replication and possibly also the decrease in CpG and UpA dinucleotides as immune stimulators. Overall, our study describes paradoxical effects of CPO of an RNA virus on viral replication and the adaptive humoral immune response.

**IMPORTANCE** Using computer algorithms and large-scale DNA synthesis, one or more ORFs of a microbial pathogen can be recoded by different strategies that involve the introduction of up to thousands of nucleotide changes without affecting amino acid coding. This approach has been used mostly to generate deoptimized viruses used as vaccine candidates. However, the effects of the converse approach of generating optimized viruses are still largely unknown. Here, various ORFs in the genome of respiratory syncytial virus (RSV) were codon pair optimized (CPO) by increasing the content of codon pairs that are overrepresented in the human genome. CPO did not affect RSV replication in multicycle replication experiments *in vitro.* However, replication was marginally reduced in two rodents models. In hamsters, CPO RSVs induced lower levels of serum RSV-neutralizing antibodies. Thus, CPO of an RNA virus for a mammalian host has paradoxical effects on virus replication and the adaptive humoral immune response.

## Abstract (precis)

Using computer algorithms and large-scale DNA synthesis, one or more ORFs of a microbial pathogen can be recoded by different strategies that involve the introduction of up to thousands of nucleotide changes without affecting amino acid coding. This approach has been used mostly to generate deoptimized viruses used as vaccine candidates. However, the effects of the converse approach of generating optimized viruses are still largely unknown. Here, various ORFs in the genome of respiratory syncytial virus (RSV) were codon pair optimized (CPO) by increasing the content of codon pairs that are overrepresented in the human genome. CPO did not affect RSV replication in multicycle replication experiments *in vitro.* However, replication was marginally reduced in two rodents models. In hamsters, CPO RSVs induced lower levels of serum RSV-neutralizing antibodies. Thus, CPO of an RNA virus for a mammalian host has paradoxical effects on virus replication and the adaptive humoral immune response.

## INTRODUCTION

Due to the degeneracy of the genetic code, most amino acids are encoded by more than one codon. Some codons are used more or less frequently than expected based on random chance. This unequal frequency of synonymous codon usage is named codon bias. Just like this unequal use of codons, some synonymous codon pairs appear in the complete set of open reading frames (ORFs) in a genome (ORFeome) more or less frequently than would be expected based the frequencies of the individual codons. This phenomenon is called “codon pair bias” (CPB) (1). It is not fully understood why some codon pairs are under- or overrepresented. It has been shown that codon pair usage affects translation due to the level of compatibility of adjacent aminoacyl-tRNAs in a translating ribosome (1, 2). It has also been suggested that CPB would be a direct consequence of dinucleotide bias (3).

The effect of CPB changes on viral biology is currently under intensive study. An increasing number of recent studies have shown that extensive recoding of viral genomes of human pathogens to increase the content of codon pairs that are underrepresented in the human ORFeome (codon pair deoptimization [CPD]) resulted in reduced virus replication *in vitro* and/or *in vivo*. This approach is a strategy to generate a new class of live-attenuated vaccine candidates (for reviews, see references 4, 5, and 6).

In contrast, potential effects of the converse strategy of extensive recoding of viral genomes using codon pairs that are overrepresented in the human ORFeome (codon pair optimization [CPO]) are still largely unknown. CPO of virus ORFs is of interest because it has the potential to specifically increase the expression of antigenic proteins, thereby increasing the host immune exposure to these antigens. The introduction of CPO ORFs also addresses the question of whether the virus in question has evolved for maximal protein expression. It is a formal possibility that suboptimal codon usage can be a viral strategy to regulate viral protein expression (7). Studies have shown that CPO of the capsid precursor region of the poliovirus genome increased protein synthesis, suggesting that CPO could enhance translation. The virulence of the resulting CPO poliovirus was unchanged or slightly increased in mice compared to that of the wild-type (wt) virus (6, 8). Unlike the case for poliovirus, CPO of the 5′-terminal region of the ORF for the vesicular stomatitis virus (VSV) polymerase L protein induced a modest attenuation in cell culture and a high level of attenuation in the mouse model (9).

In the present study, we investigated the effects of CPO of various regions of the genome of respiratory syncytial virus (RSV), representing in aggregate 9 of the 11 RSV ORFs. RSV is a major worldwide human respiratory pathogen, having greatest impact in the pediatric and elderly populations. An approved vaccine or antiviral drug suitable for routine use is not yet available. RSV belongs to the *Pneumoviridae* family of the *Mononegavirales* order. Its genome is a single-stranded negative-sense 15.2-kb RNA carrying 10 genes in the order 3′-NS1-NS2-N-P-M-SH-G-F-M2-L-5′, preceded by a short leader region and followed by a short trailer region. The M2 mRNA encodes two proteins, M2-1 and M2-2, expressed from overlapping ORFs. The genes are each flanked by short gene start and gene end transcription signals and are transcribed as individual mRNAs by sequential transcription initiating at a single promoter in the leader region. As is typical for *Mononegavirales*, RSV transcription has a polar gradient in which gene transcription decreases with increasing distance of viral genes from the leader region. This gradient occurs because some of the polymerase complexes disengage from the genomic RNA at the various gene junctions (10).

RSV in particular is an interesting viral model to evaluate the effect of large-scale CPO on viral biology because we previously showed that RSV could tolerate extensive CPD of most of its ORFs (11), whereas extensive CPD in other viruses appeared to result in excessive restriction of virus replication (6, 12). Indeed, in our previous study (11), CPD of 9 of the 11 RSV ORFs by introduction of a total of 2,692 mutations was attenuating, but not excessively so. In the present work, 4 CPO RSV genomes were designed, synthesized, and rescued by reverse genetics. Because of the possibility of a resulting increase in the replication efficiencies of these viruses, CPO was done using an RSV backbone that was attenuated by 2 mutations in the L gene, namely, the codon deletion Δ1313 mutation and the missense mutation I1314L (each numbered according to the codon in the L ORF). The Δ1313 mutation confers temperature sensitivity (ts) and attenuation for replication *in vivo* (13). The I1314L mutation genetically stabilizes the Δ1313 mutation against deattenuation. The presence of this stabilized attenuating mutation thus provided safety against potentially increased virulence. The effects of CPO of RSV ORFs on virus biology were investigated.

## RESULTS

### Design of CPO rRSVs.

**FIG 1:**
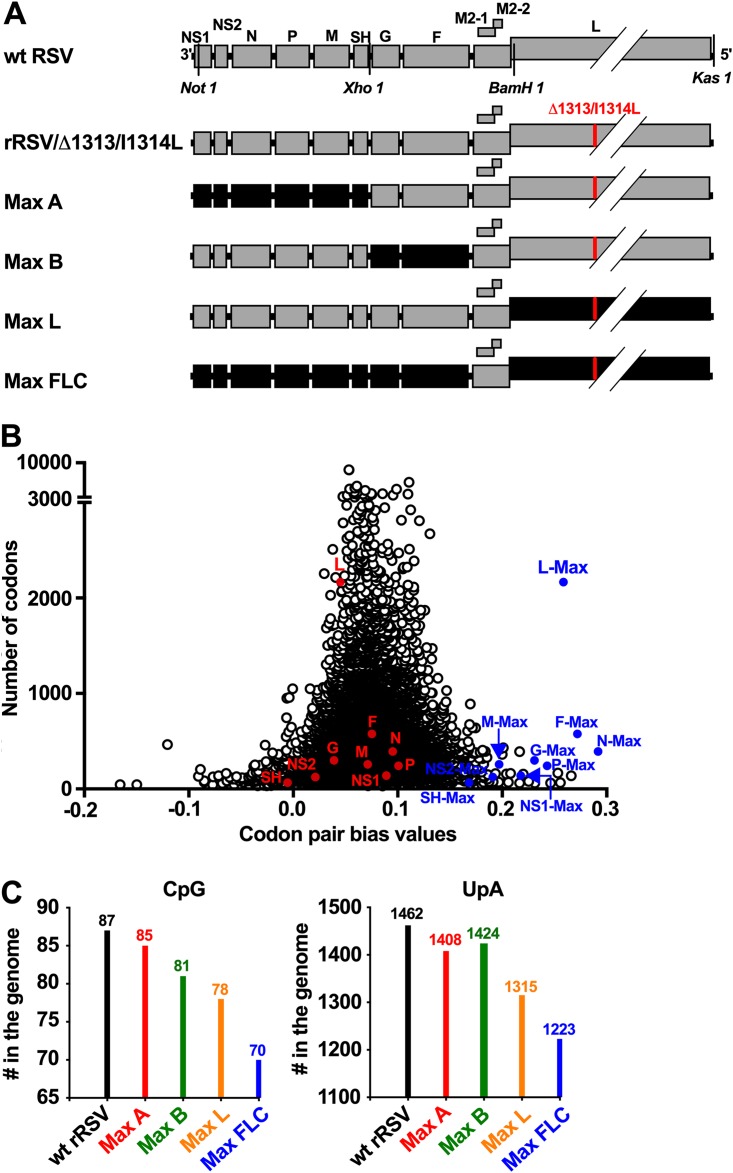
Generation of codon pair-optimized (CPO) rRSVs. (A) Gene maps of the CPO rRSVs Max A, Max B, Max L, and Max FLC. The wt and CPO ORFs are shown as gray and black boxes, respectively. Restriction sites used for the constructions are indicated. The red bar in the L ORF indicates the positions of the Δ1313 codon deletion and I1314L missense mutations. For simplicity, “Δ1313/I1314L” was not included in the names of the CPO viruses. (B) Codon pair bias (CPB) values plotted against ORF lengths in codons, shown for wt RSV ORFs (red circles), CPO RSV ORFs (blue circles), and a set of 14,795 National Center for Biotechnology Information (NCBI)-annotated human protein-coding sequences (Consensus Coding Sequence [CCDS] database published March 2005) (open circles). As described previously (1, 6), the CPB score for each ORF is the mean of the codon pair scores (CPSs) for all of its codon pairs. In turn, the CPS for each codon pair is the natural log of the ratio of the observed versus expected frequency of that codon pair in the human ORFeome. (C) Total numbers of CpG and UpA dinucleotides in the wt and CPO viral genomes.

We used gene synthesis and reverse genetics to create 4 rRSVs, named Max A, Max B, Max L, and Max FLC (Fig. 1A), in which, respectively, 6, 2, 1, and 9 of the 11 RSV ORFs were recoded using a computer algorithm to achieve the most positive CPB based on usage in the human ORFeome (Fig. 1B). The patterns of ORFs subjected to codon pair optimization (CPO) in these four viruses were the same as were chosen for CPD in our previous study (11). This large-scale recoding by codon pairs overrepresented in the human ORFeome was done with no changes to amino acid coding and included only a small number of post-CPO manual changes in synonymous codon usage to remove excessive homopolymer tracts and sequences resembling RSV *cis*-acting signals that were introduced incidentally by CPO. The algorithm used in the CPO was designed to leave the predicted free energy of RNA folding unchanged. However, the RSV genome naturally contains a number of homopolymer tracts of 6 or 7 nucleotides (nt) in length (specifically, 27 U tracts, 5 A tracts, and 1 G tract, with no C tracts), and the process of CPO interrupted most of these homopolymeric tracts (specifically, the tracts that were interrupted were as follows: 21 U homopolymers in the wt NS1, NS2, N, P, M, G, F, and L ORFs, an A homopolymer in the wt F ORF, two A homopolymers in the wt L ORF, and the sole G homopolymer in the wt L ORF). However, it is not know whether this has any consequence for the viruses. The M2-1 and M2-2 ORFs were not subjected to CPO because these overlapping ORFs engage in coupled stop-start translation that is thought to depend on sequence, and possibly secondary structure, located in the M2-1 ORF approximately 150 nt upstream of the M2-2 ORF (14). Given the incompletely defined nature of the *cis*-acting element(s) in the M2 gene, we left it undisturbed. As a further consideration, the second ATG (codon 48) of the G ORF of wt RSV normally serves as a second translational initiation site that gives rise to an abundant N-terminally truncated form of G that is secreted (sG). In the CPO G ORF, recoding changed only one nucleotide assignment (position −4, G → T) in the 12 positions immediately upstream and the six immediately downstream of this ATG. For eukaryotic mRNAs in general, the −4 position has little influence in translational initiation, and there is little difference between G and T (reference 15 and references cited therein). Therefore, based on these well-established rules, the CPO described here should have little or no effect on translational initiation of sG. Finally, because CPO of the viral ORFs might increase viral fitness, each of the CPO viruses was constructed to be attenuated by the introduction of 2 mutations, namely, the deletion of codon 1313 in the L ORF (Δ1313 mutation) and the missense mutation I1314L, but for the sake of simplicity these mutations are not included in the viral names. The CPO viruses were compared to unmodified wt recombinant RSV (rRSV) as well as to wt rRSV bearing the Δ1313/I1314L mutations (designated rRSV/Δ1313/I1314L), the latter being the direct non-CPO comparator for the CPO viruses.

The effect of the CPO on the CPB of each ORF is shown in Fig. 1B. Whereas the ORFs of wt RSV have CPB values comparable to those of the human ORFeome, those of the CPO RSV ORFs were much higher, illustrating the high content of overrepresented codon pairs. The nucleotide sequence identity between the wt and CPO RSV ORFs ranged from 78% (F) to 89% (SH), and the number of nucleotide differences increased with increasing ORF length and ranged from 21 (SH) to 1,315 (L) (Table 1). The percentage of C+G and A+U nucleotides was largely unchanged (not shown). However, the number of CpG and UpA dinucleotides, which are potential inducers of host immunity (16, 17), decreased with increasing extent of CPO (Fig. 1C). For example, 87 CpG dinucleotides and 1,462 UpA dinucleotides were present in the wt sequence, while 70 CpG dinucleotides and 1,223 UpA dinucleotides were present in the Min FLC sequence. These reductions are 19.5% and 16.3% decreases in the numbers of CpG and UpA dinucleotides, respectively.

**TABLE 1:** Percent nucleotide identity and number of substitutions between wt and CPO RSV ORFs

ORF	% identity	No. of substitutions
NS1	85.7	60
NS2	82.7	65
N	81.5	217
P	80.3	143
M	83.7	126
SH	89.2	21
G	81.5	166
F	78.3	375
L	79.8	1,315

### CPO of RSV ORFs had minimal effects on virus replication *in vitro*, ts phenotype, or specific infectivity.

**FIG 2:**
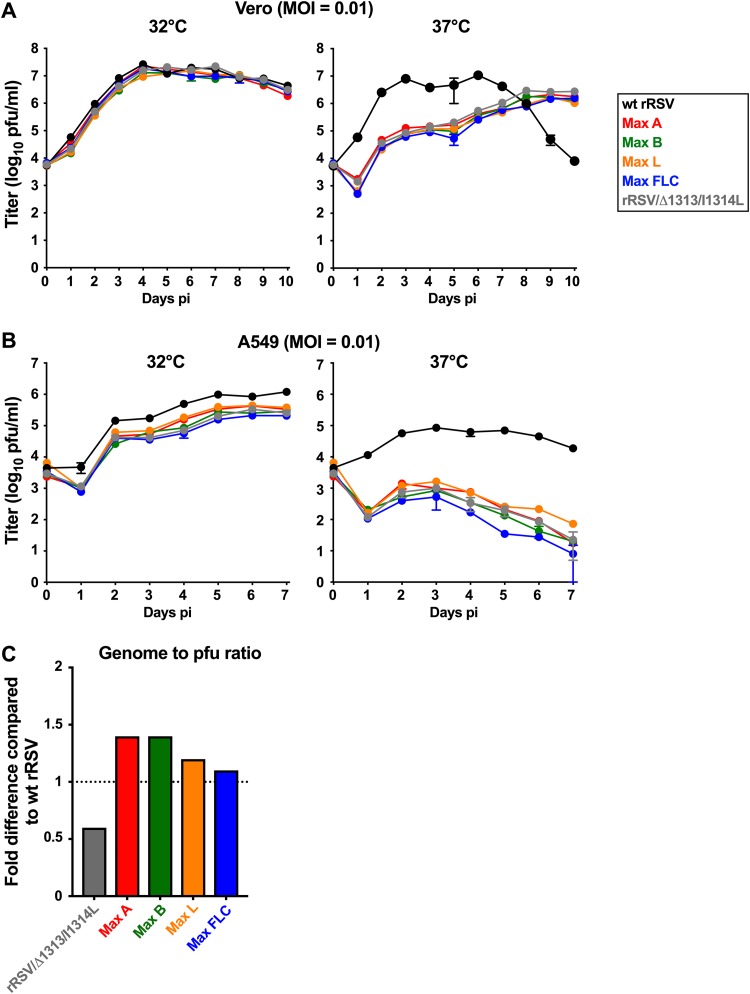
Replication of CPO rRSVs and specific infectivity (genome-to-PFU ratio) of virus stocks. (A and B) The multicycle growth kinetics of wt rRSV, rRSV/Δ1313/I1314L, and the CPO rRSVs were assessed in Vero (A) and A549 (B) cells. Replicate cell monolayers were infected with wt rRSV, rRSV/Δ1313/I1314L, or the CPO rRSVs at a multiplicity of infection (MOI) of 0.01 and incubated at 32°C (left) or 37°C (right). Duplicate monolayers per virus were harvested daily from day 1 to 10 (Vero cells) or day 1 to 7 (A549 cells): the cells were scraped into the medium and vortexed for 30 s to release cell-associated virus, clarified cell culture medium supernatants were flash-frozen, and later titers were determined in parallel by immunoplaque assay in duplicate in Vero cells at 32°C. The results are expressed as mean values with standard deviations (SD) (note that for most time points, the SD value is so small that the its bars are not distinct from the filled circle marking the time point). The MOIs of the inocula also were confirmed by titration. (C) Specific infectivity. The genome-to-PFU ratios of CPO rRSVs, compared to those of wt rRSV and rRSV/Δ1313/I1314L, were evaluated by RT-qPCR. RNA was extracted from virus preparations, aliquots representing approximately 2 × 10^4^ PFU of each virus were subjected to RT using a primer in the M2 ORF specific to genomic (negative-sense) RNA, and then 10% of each cDNA reaction mixture was amplified by a genome-specific qPCR. The results are expressed as fold difference compared to wt rRSV.

Each of the CPO rRSVs was readily recovered from cDNA, and the genome sequences of the recovered viruses were analyzed and confirmed to be free of adventitious mutations. We evaluated multicycle replication of the CPO viruses in African green monkey kidney Vero and human lung epithelial A549 cell lines inoculated with a multiplicity of infection (MOI) of 0.01 and incubated at 32°C or 37°C (Fig. 2).

In Vero cells at 32°C, the growth of each of the CPO viruses reached a maximum titer of approximately 10^7^ PFU/ml on day 4, and the kinetics and titers were indistinguishable from those of wt rRSV and rRSV/Δ1313/I1314L (Fig. 2A). wt rRSV replicated faster at 37°C than at 32°C, with the peak titer at day 3; however, the peak titers were similar at the two temperatures. Max A, Max B, Max L, Max FLC, and rRSV/Δ1313/I1314L exhibited reduced replication at 37°C (around 100-fold lower than that of wt rRSV at day 3), consistent with the presence of the ts Δ1313/I1314L mutation in each virus. None of the CPO viruses replicated differently than rRSV/Δ1313/I1314L, indicating that the reduced replication at 37°C likely was due completely to the presence of the Δ1313/I1314L mutation.

In A549 cells at 32°C (Fig. 2B), all four CPO viruses replicated with similar kinetics and to similar maximum titers as rRSV/Δ1313/I1314L, which were slightly reduced compared to the case for wt rRSV. At 37°C, replication of the CPO viruses and rRSV/Δ1313/I1314L was strongly restricted compared to that of wt rRSV, but they were similar to each other. As with Vero cells, this indicated that the restriction was due to the Δ1313/I1314L mutation. Taken together, these data showed that CPO of RSV ORFs did not affect the kinetics or yield of multicycle viral replication at 32°C or 37°C in cells that are competent (A549) or not competent (Vero) to mount a type I interferon (IFN) response to viral infection.

We previously found that each of the four CPD RSVs that were made, representing in aggregate nine different ORFs, had a ts phenotype (11). Therefore, we investigated the effect of CPO of RSV ORFs on the viral ts phenotype by evaluating the ability of CPO viruses to form plaques on Vero cells at temperatures ranging from 32 to 40°C (Table 2). wt rRSV was not ts at physiological temperatures, as expected. The shutoff temperature (*T*_SH_) (the lowest restrictive temperature at which there is a reduction in plaque number compared to that at 32°C that is ≥100-fold that observed for wt RSV at the two temperatures [Table 2]) of Max A, Max B, and Max L was 39°C and was identical to that of rRSV/1313/I1314L. Max FLC had a *T*_SH_ of 38°C, indicating that CPO decreased its *T*_SH_ by 1°C. These data showed that CPO of RSV ORFs conferred the ts phenotype only in the case of Max FLC and in that case only marginally.

**TABLE 2:** Temperature sensitivity of the CPO RSVs on Vero cells

Virus	Δ1313/I1314L introduced	Virus titer (log_10_ PFU/ml) at indicated temp (°C)^*a*^							*T*_SH_ (°C)
		32	35	36	37	38	39	40	
Max A	Y	7.8	7.7	7.7	7.5	6.5	2.2	<1	39
Max B	Y	7.6	7.6	7.5	7.2	6.4	1.7	<1	39
Max L	Y	7.8	7.8	7.6	7.3	6.1	2.0	<1	39
Max FLC	Y	7.7	7.6	7.4	7.1	5.3	1.7	<1	38
rRSV/Δ1313/I1314L	Y	7.8	7.7	7.6	7.4	6.6	2.5	<1	39
wt rRSV	N	7.6	7.8	7.7	7.7	7.7	7.6	7.6	>40

a The ts phenotype for each virus was evaluated by assessing virus growth on Vero cells at the indicated temperatures utilizing temperature controlled water baths (26). For viruses with a ts phenotype, the shutoff temperature (*T*_SH_) is underlined. The *T*_SH_ is defined as the lowest restrictive temperature at which there is a reduction in plaque number compared to that at 32°C that is 100-fold or greater than that observed for wt RSV at the two temperatures. The ts phenotype is defined as a *T*_SH_ of 40°C or less.

We also evaluated the specific infectivity of wt rRSV, rRSV/Δ1313/I1314L, and the CPO rRSVs by measuring the ratio of genomic RNA to PFU in virus preparations using quantitative reverse transcription-PCR (RT-qPCR) for the M2 gene, whose sequence was unchanged and identical in all of the viruses (Fig. 2C). There were only minor differences between some of the viruses in the genome-to-PFU ratio, indicating that CPO had no major effect on virus-specific infectivity.

### Effects of CPO on the production of viral RNA during single-cycle replication.

**FIG 3:**
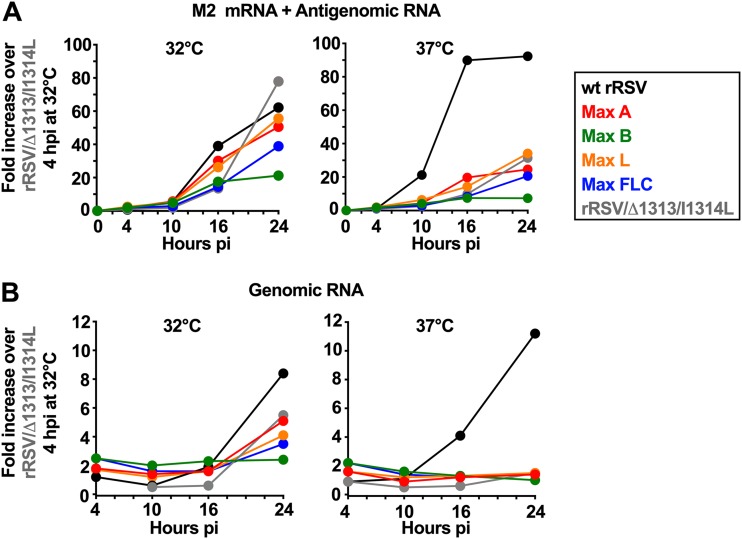
Effects of CPO on the production of RSV RNAs during single-cycle replication of wt rRSV, rRSV/Δ1313/I1314L, and the CPO rRSVs. Replicate wells of Vero cell monolayers were infected at an MOI of 3 PFU per cell with the indicated viruses or mock infected and then incubated at 32°C or 37°C as indicated. At 4, 10, 16, and 24 hpi, one well per virus was harvested and processed for analysis of cell-associated RNA by strand-specific RT-qPCR, with primers and probe specific to the M2 gene, to detect positive-sense RSV RNA (mRNA and antigenome) (A) or negative-sense RSV RNA (genome) (B). Results were normalized to 18S rRNA. The M2 gene was used for comparison of all viruses because it was the only gene that remained unchanged in the wt and CPO rRSVs. Data are expressed as fold increase over rRSV/Δ1313/I1314 at the 4-h time point.

**FIG 4:**
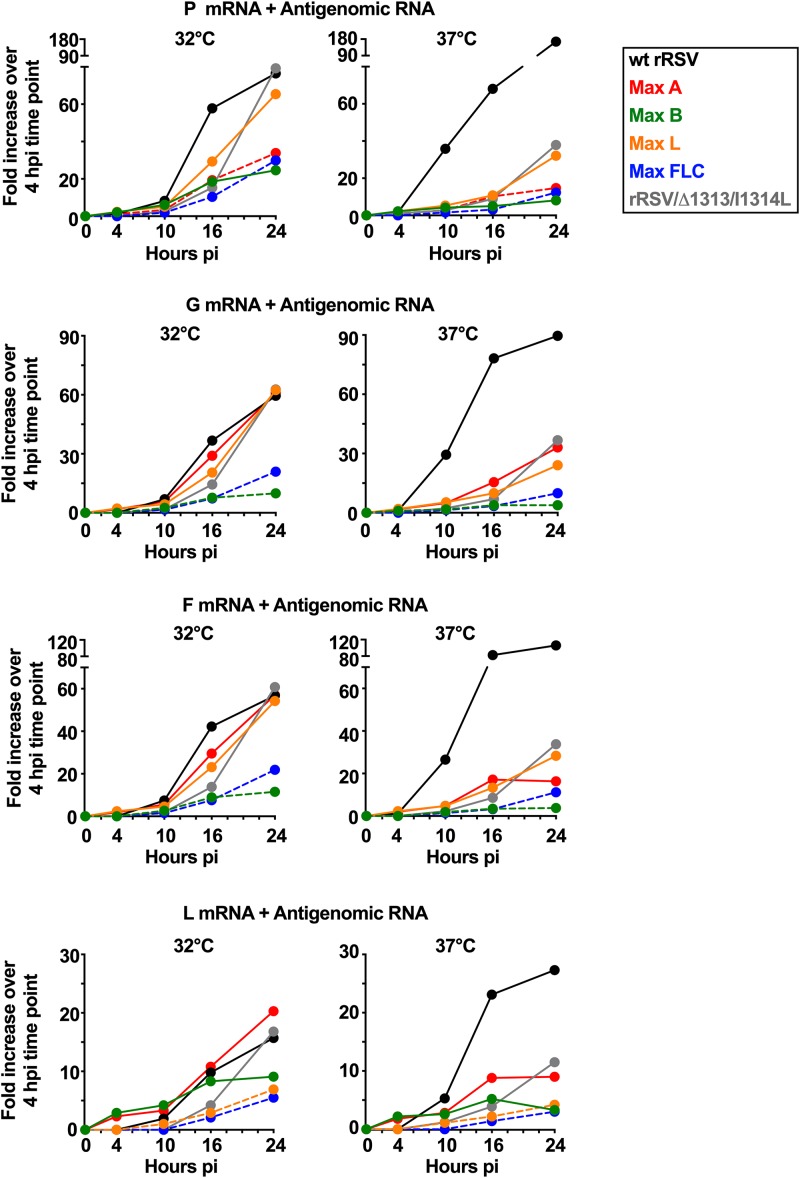
Effects of CPO on the production of positive-sense RSV P, G, F, and L RNAs (mRNA plus antigenome) during single-cycle replication at 32°C and 37°C. This is a continuation of Fig. 3A, which showed RT-qPCR quantification of the expression of the M2 mRNA during infection of Vero cells at 32°C with wt rRSV, rRSV/Δ1313/I1314L, and Max derivatives. The corresponding data for the P, G, F, and L mRNAs at 32°C and 37°C are shown here. Quantification of cell-associated RNA was done by positive-sense strand-specific RT-qPCR, with results normalized to 18S rRNA. As described in Materials and Methods, the extensive sequence differences between the wt and CPO versions of the P, G, F, and L ORFs necessitated the use of two different sets of primers/probes for wt rRSV versus CPO ORFs. Even though TaqMan assays were selected for optimal efficiency, direct comparison of relative abundances at the different time points were made among viruses that had the same sequence of the ORF of interest. In addition, data are expressed as fold increase over the result for rRSV/Δ1313/I1314 at the 4-h time point for wt genes (solid lines) and as fold increase over the result for Max FLC at the 4-h time point for CPO genes (dotted lines).

We evaluated the production of cell-associated viral RNA in Vero cells infected at an MOI of 3 PFU/cell with wt and CPO rRSVs in a single-cycle replication experiment (Fig. 3 and 4). First, the RNA was quantified by RT-qPCR using primers and probes for the M2 gene that were specific to RSV positive-sense RNA (M2 mRNA and antigenomic RNA) (Fig. 3A) or negative-sense RNA (genomic RNA) (Fig. 3B). Because the M2 gene had been left unchanged in the CPO viruses, this allowed direct comparison between all of the viruses.

The accumulation of cell-associated positive-sense M2 RNA by wt rRSV at 32°C was detected by 10 h postinfection (hpi) and was maximal between 16 and 24 hpi (Fig. 3A). In comparison, the CPO viruses and rRSV/Δ1313/I1314L exhibited delayed and decreased production of positive-sense M2 RNA. The production of positive-sense RNA by Max A and Max L was greater than that by rRSV/Δ1313/I314L at 16 hpi, although this difference was gone by 24 hpi (Fig. 3A). In comparison, the production of positive-sense RNA by Max FLC and Max B was similar to that by rRSV/Δ1313/I314L at 16 hpi but thereafter was less (Fig. 3A). For wt rRSV, the accumulation of positive-sense RNA at 37°C was faster and increased compared to that at 32°C. For the CPO viruses and rRSV/Δ1313/I1314L, it was slower and reduced.

The production of cell-associated genomic RNA for wt rRSV at 32°C, assayed with M2-specific tagged primers and probe (Fig. 3B), was detected by 16 hpi and increased until 24 hpi. Accumulation of genomic RNA with Max A, Max L, or Max FLC was detected at 24 hpi and overall was similar to that of rRSV/Δ1313/I1314L. An increase in accumulation of genomic RNA was not detected with Max B at 32°C (Fig. 3B) and was not detected at 37°C for any CPO virus or rRSV/Δ1313/I1314L.

We also quantified the accumulation of cell-associated positive-sense P, G, F, and L RNAs at 32°C and 37°C (Fig. 4). The extensive sequence differences between the wt and CPO ORFs necessitated the use of two different sets of primers/probes for ORFs with wt sequence versus CPO sequence. Even though all TaqMan assays were selected for optimal efficiency, this limited the comparison of relative abundances for viruses bearing wt versus CPO versions of a given ORF. Furthermore, expression of the wt ORFs was normalized to rRSV/Δ1313/I1314L, while that of the CPO ORFs was normalized to Max FLC.

In general, when the expression of positive-sense RNA for wt P, G, F, and L ORFs was compared at 10 to 16 hpi at 32°C among those viruses bearing these ORFs in wt form, expression of wt RSV generally was greater, followed by Max A, Max L, and rRSV/Δ1313/I1314L (Fig. 4, left panels). However, by 24 hpi these viruses generally were similar. Thus, expression from CPO viruses such as Max A and Max L often was somewhat greater than that of compared to rRSV/Δ1313/I1314L at 10 to 16 hpi. In contrast, expression of wt ORFs from Max B was low at all time points. Expression of CPO ORFs was generally low at all time points. Whether this low expression reflects differences in synthesis, stability, or the RT-qPCR assay is unknown.

At 37°C (Fig. 4, right panels), the viruses bearing the ts attenuating Δ1313/I1314L mutations were reduced more severely than wt rRSV, as would be expected for viruses bearing a ts mutation. In general, the rRSV/Δ1313/I1314L, Max A, and Max L viruses produced more RNA than the Max FLC and Max B viruses. The observation that RNA production generally was lower for the Max FLC and Max B viruses under all conditions suggested that CPO of the G and/or F ORFs (which is a common feature of these two CPO viruses and not the other two CPO viruses) reduced overall gene expression.

### Effects of CPO on protein expression during single-cycle replication.

**FIG 5:**
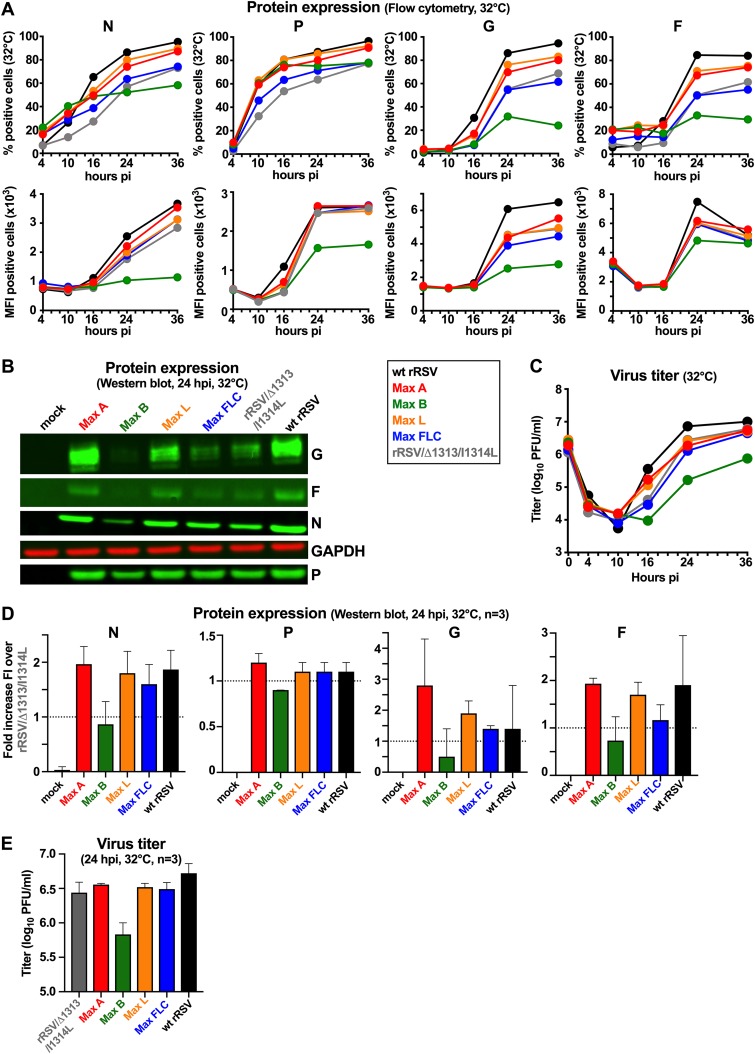
Effects of CPO on the production of RSV proteins and virions during single-cycle replication of wt rRSV, rRSV/Δ1313/I1314L, and CPO rRSVs. (A to C) Additional replicate wells of Vero cell monolayers were infected in parallel with those for Fig. 3, incubated at 32°C or 37°C, and harvested for analysis by flow cytometry (A), Western blotting (B), and titration (C) to measure virus production. (A) Flow cytometry. One well per virus/time point, from cells incubated at 32°C, was harvested at 4, 10, 16, 24, and 36 hpi and analyzed by flow cytometry to determine the percentage of cells that expressed detectable N, P, G, and F proteins as well as the median fluorescence intensity (MFI) of the positive cells. (B) Western blot analysis. One well per virus, incubated at 32°C, was harvested at 24 hpi and analyzed by Western blotting. The G, F, N, and P proteins are shown, with GAPDH as a loading control. (C) Virus production. One well per virus/time point was harvested at 0, 4, 10, 16, 24, and 36 hpi and processed for virus titration as described for Fig. 2. (D and E) Two additional single-cycle replication experiments involving Western blot analysis and virus production were performed using the same conditions as for panels B and C, respectively, to provide confirmatory data. (D) Western blotting. For each of two additional experiments, one well per virus, incubated at 32°C, was harvested at 24 hpi and analyzed by Western blotting as for panel B. The data from these two experiments were combined with those from panel B and are expressed as fold increase in fluorescence intensity (FI) over that of rRSV/Δ1313/I1314L at the same time point, with the range shown. (E) Virus production. For each of two additional experiments, one well per virus/time point, from cells incubated at 32°C, was harvested at 24 hpi and processed for virus titration as described for panel C. The data from these two experiments were combined with those from panel C and are expressed as median values with the range shown.

From the same experiment used for Fig. 3 and 4, we also evaluated the production of cell-associated viral N, P, G, and F proteins in Vero cells at 32°C by flow cytometry (Fig. 5A) and Western blot analysis (Fig. 5B). When analyzed by flow cytometry (Fig. 5A), the increase of infected cells over time (based on the percentage of cells positive for expression of N, P, G, or F protein) was greatest for wt rRSV, followed in order of decreasing abundance by Max A and Max L (which were similar to each other), rRSV/Δ1313/I1314L and Max FLC (which were similar to each other), and Max B. Our interpretation is that, even though these viruses have essentially the same specific infectivity and were infected at the same MOI, antigen-positive cells were detected at a lower rate for viruses such as Max B because of a reduced rate of protein expression. In general, the kinetics of G and F expression lagged behind those of N and P expression. While a strong increase in N and P expression was detected for all viruses within the first 16 h after infection, G and F glycoprotein expression strongly increased from 16 to 24 hpi. Interestingly, at 36 h after infection, fewer than 50% of Max-B infected cells that expressed N and P also had G and F expression detectable by flow cytometry. In addition, as shown by the measure of the median fluorescence intensity (MFI) of the protein-expressing cells, Max B-infected cells expressed about 2 to 3 times less N, P, and G proteins than cells infected with the other viruses. These data confirmed that CPO of G and F strongly decreased expression of these proteins in RSV-infected cells. The percentage of Max FLC-infected cells that expressed those proteins was intermediate between Max A or Max L and Max B, suggesting that the positive effect of the CPO on genes in Max A and Max L was counterbalanced by the negative effect of the CPO of F and/or G.

Western blot analysis of RSV protein expression from the same experiment (Fig. 5B) as well as two additional experiments (Fig. 5D) was consistent with the flow cytometry data (Fig. 5C). In particular, production of the G and F proteins was increased in Max A and Max L (2- to 3-fold increase) compared to rRSV/Δ1313/I1314L, while protein expression was strongly reduced in Max B-infected cells and was intermediate in Max FLC-infected cells.

### Effects of CPO on virus production during single-cycle replication.

In the same single-cycle replication experiment used for Fig. 3, 4, and 5A and B, we also evaluated the production of virus particles (Fig. 5C). The production of infectious wt rRSV was first observed at 16 hpi at 32°C and 37°C (Fig. 5C and data not shown), which is approximately concurrent with the accumulation of genomic RNA (Fig. 3B) and glycoprotein expression (Fig. 5A). Replication of the CPO rRSVs and rRSV/Δ1313/I1314L was reduced at 37°C, as would be expected due to the presence of the ts mutation (data not shown). At 32°C, replication of Max A, Max L, and Max FLC was comparable to that of rRSV/Δ1313/I1314L and wt rRSV. Replication of Max B was reduced approximately 10-fold compared to that of the other viruses. The data obtained at 24 hpi in this experiment were confirmed in two additional independent experiments (Fig. 5E).

### Expression of the G and F proteins from wt and CPO ORFs contained in minigenomes.

**FIG 6:**
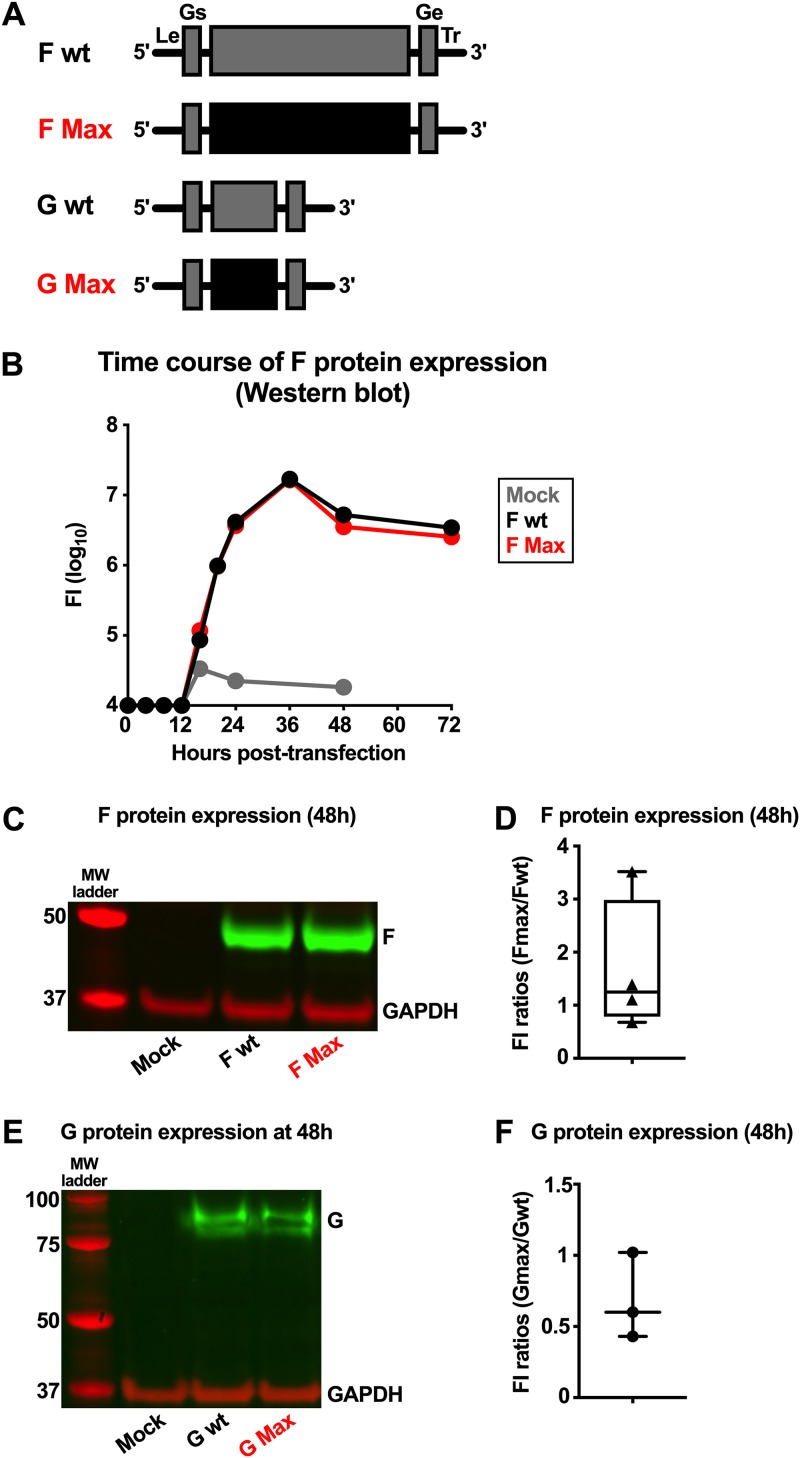
Expression of G and F proteins from wt and CPO ORFs contained in minigenomes. Four RSV minigenomes were constructed that each contained a single gene with a wt or CPO ORF encoding RSV G or F. (A) Gene maps of the four cDNAs used to evaluate the expression of F and G. Each cDNA contained a single G or F ORF, either wt or CPO, under the control of wt G or wt F gene start (Gs) and gene end (Ge) transcription signals, with an upstream RSV leader region (Le) and downstream trailer region (Tr). Each cDNA was cloned into a pBluescript plasmid vector, with the Le region preceded by a T7 promoter and the Tr region followed by a self-cleaving ribozyme (not shown), such that expression by the T7 RNA polymerase yielded a positive-sense RNA copy with correct 3′ and 5′ ends. (B to F) The ability of the CPO versus wt F and G ORFs to express F and G was tested on BSR T7/5 cells that constitutively express the T7 RNA polymerase. BSR T7/5 cells were transfected with a plasmid mixture encoding the indicated minigenome, together with the four RSV support plasmids expressing the N, P, M2-1, and L proteins, which are necessary to reconstitute the virus polymerase complex that directs viral transcription and RNA replication. Mock-transfected cells were used as controls. Cells were incubated at 32°C and harvested at the indicated times for analysis by Western blotting. (B) Kinetics of F protein expression, evaluated by Western blotting and expressed as fluorescence intensity (FI). (C and D) Western blots to evaluate F protein expression at 48 h posttransfection, with GAPDH as a loading control and a protein ladder as markers for protein size. (C) Results from a representative experiment. (D) The FI of F protein from CPO versus wt ORFs from 4 independent Western blot experiments using dye-labeled antibodies was measured as described in Materials and Methods. Data are represented as F Max/F wt ratios. The box plots show the median (horizontal line) flanked by the second and third quartiles. The outer bars show the range of values. (E and F) Western blots to evaluate G protein expression. (E) Results from a representative experiment. (F) FI ratios of G protein from CPO versus wt ORFs from 3 independent experiments. The median (horizontal line) and the range of values are shown.

The very strong restriction of RNA and protein synthesis and viral replication observed for Max B raised the possibility that the CPO G and/or F ORF might coincidentally contain an unintended inhibitory sequence. This sequence could have interfered with protein synthesis, viral transcription, or genome replication, perhaps through some effect in addition to the CPO. To investigate this possibility, four RSV minigenomes were chemically synthetized *de novo* (GenScript). Each contained a single ORF encoding wt or CPO G or F protein (Fig. 6A) flanked 5′ by a T7 promoter, the viral leader region, and a gene start transcription signal and flanked 3′ by a gene end transcription signal, the viral trailer region, and a self-cleaving ribozyme cloned into a pBluescript plasmid vector. The minigenome sequences were completely confirmed by Sanger sequencing. Transcription by T7 RNA polymerase yielded a positive-sense copy of the minigenome.

BHK-T7 cells that constitutively express the RNA T7 polymerase were transfected with each construct as well as with the RSV support plasmids expressing the viral N, P, M2-1, and L proteins required for viral transcription and genome replication. Cells were incubated at 32°C and harvested at the indicated times for analysis by Western blotting. G and F protein expression was evaluated (Fig. 6B to F). Under these conditions, the expression of CPO G and F proteins was comparable to that of wt G and F, suggesting that these sequences were properly recognized by the virus replication complex and did not exhibit obvious intrinsic defects that could explain the reduced replication of Max B. It also suggested a lack of substantially increased synthesis of the G and F proteins in response to CPO. In experiments in which the RSV L support plasmid was omitted, expression of the RSV G and F proteins was undetectable (data not shown), confirming that the substantial levels of expression of the G and F proteins by these minigenomes were dependent on transcription and replication by the RSV transcription/replication complex.

### CPO of RSV genes caused a marginal reduction in virus replication *in vivo* but strongly reduced the antibody response.

**FIG 7:**
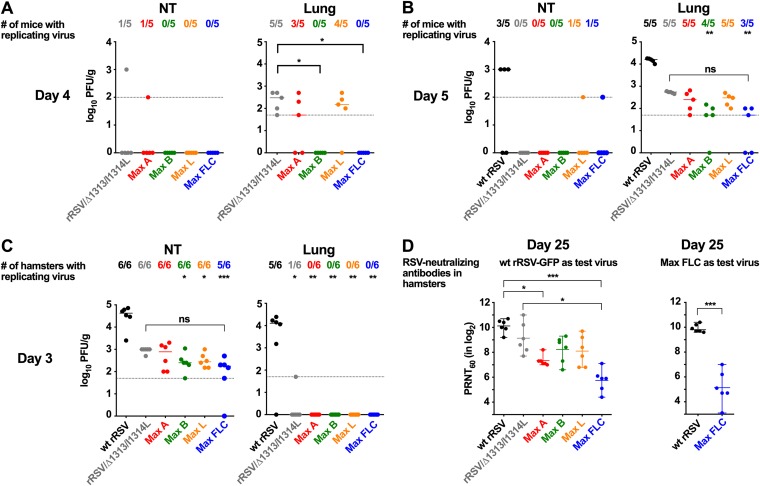
Replication and immunogenicity of CPO rRSVs in rodents. Replication of CPO viruses was evaluated in the respiratory tracts of 6-week-old BALB/c mice (A and B) and 6-week-old golden Syrian hamsters (C), and immunogenicity was investigated in hamsters (D). (A to C) Groups of 10 mice (5 mice for wt rRSV) and 12 hamsters were inoculated i.n. with 10^6^ PFU of the indicated virus per animal. On days 3 (hamsters) (C), 4 (mice) (A), and 5 (mice) (B), virus titers in the nasal turbinates (NT) and lungs were determined. The limits of virus detection, indicated by dotted lines, were 100 and 50 PFU/g for the NT and lung specimens of mice, respectively, and 50 PFU/g for the NT and lung specimens of hamsters. The number of animals per group with detectable virus is indicated along the top. (D) Serum RSV-neutralizing antibodies at day 25 in hamsters (6 hamsters per group). The 60% plaque reduction neutralizing antibody titers (PRNT_60_) were determined using wt rRSV-GFP (left panel) or Max FLC (right panel), as indicated. In panel A (right panel), brackets labeled with asterisks compare rRSV/Δ1313/I1314L with Max B or Max FLC. Note there are no data for wt rRSV in panel A. In panels B (right panel) and C (left panel), statistical differences for several viruses versus wt rRSV are indicated by an asterisk(s) located under the number of mice or hamsters with replicating virus. In addition, in panels B (right panel) and C (left panel), brackets indicate a lack of significant difference (nonsignificant [ns]) between rRSV/Δ313/I1314L and Max FLC. In panel D, brackets labeled with asterisks indicate the significance of differences between wt rRSV or rRSV/Δ1313/I1314L and the indicated viruses *, *P* ≤ 0.05; **, *P* ≤ 0.01; ***, *P* ≤ 0.001.

We next evaluated replication in mice and hamsters and immunogenicity in hamsters of the CPO viruses compared to wt rRSV following intranasal (i.n.) inoculation (Fig. 7).

In the nasal turbinates (NT) of mice, wt rRSV was detected in 3 out of 5 animals on day 5 (median titer of 10^3^ PFU/g), whereas the CPO viruses and rRSV/Δ1313/I1314L were detected only sporadically (Fig. 7B). In the lungs, wt rRSV was detected in all of the animals, and the titers reached 10^4^ PFU/g on day 5. In comparison, rRSV/Δ1313/I1314L also was detected in all of the animals, but its titer was 30-fold lower on day 5. The titers of Max A and Max L were slightly reduced compared to those of rRSV/Δ1313/I1314L on days 4 and 5, but the difference was not significant (Fig. 7A and B). Max B and Max FLC were not detected on day 4, and on day 5 their titers were reduced approximately 10-fold compared to that of rRSV/Δ1313/I1314L. These data suggested that CPO of the F and G ORFs (a feature of Max B and Max FLC but not of Max A or Max L) was associated with a significant reduction in RSV replication in mice, while CPO of other ORFs caused only a minor reduction.

We next evaluated the replication of CPO rRSVs compared to wt RSV in the respiratory tract of hamsters on day 3 postinoculation (Fig. 7C). wt rRSV replicated efficiently in the upper and lower respiratory tracts of hamsters, with a median titer of 10^5^ or 10^4^ PFU/g in the NT or lungs, respectively. Replication of rRSV/Δ1313/I1314L was reduced approximately 100-fold in the NT and was almost completely abolished in the lungs, with only 1 of 6 animals having detectable virus. Replication of Max B, Max L, and Max FLC was reduced approximately 5-fold in the NT compared to that of rRSV/Δ1313/I1314L, but this effect was not significant. None of the CPO viruses were detected in the lungs. Overall, the replication of the CPO RSVs in hamsters was only marginally reduced compared to that of rRSV/Δ1313/I1314L.

We also measured the serum RSV-neutralizing antibody response at day 25 postinfection (p.i.) in hamsters (Fig. 7D). rRSV/Δ1313/I1314L induced an antibody response that was strong but reduced compared with that of wt rRSV, probably due to its temperature sensitivity and reduced replication in the lower respiratory tract, but the difference was not significant. Surprisingly, each of the CPO rRSVs induced a reduced antibody response compared to rRSV/Δ1313/I1314L. This effect was more apparent for Max A and Max FLC (*P* < 0.05 compared with rRSV/Δ1313/I1314L). This result showed that CPO of RSV ORFs reduced the antibody response. It suggested that this effect was increased with an increased number of CPO ORFs (e.g., Max A and Max FLC) and was not strictly proportional to the level of replication.

We next asked if the low neutralizing antibody titer detected in the sera of Max FLC-infected hamsters in particular might somehow be an artifact of the use of wt rRSV-green fluorescent protein (GFP) as the test virus in the neutralization assay, rather than the fully homologous Max FLC virus. To answer that question, we measured the neutralizing antibody response in the sera of wt rRSV- and Max FLC-infected hamsters using Max FLC as the virus in the neutralization assay. This comparison confirmed that the neutralizing antibody titer in the sera of Max FLC-infected hamsters indeed was significantly lower (*P* < 0.001) than that in the sera of wt rRSV-infected hamsters. Thus, the antibody titer induced by Max FLC really was strongly reduced compared to that induced by rRSV/Δ1313/I1314L.

On day 29, hamsters were challenged i.n. with wt rRSV, and NT and lungs were harvested at 3 days postchallenge. No detectable challenge virus replication was detected in any animal (not shown). Thus, even the relatively reduced immune responses induced by some of the viruses were sufficient to completely restrict challenge RSV replication in this semipermissive model.

We investigated whether the CPO virus preparations contained defective genomes, which might reduce virus replication and/or increase immunogenicity (18, 19). To do so, viral RNA from each virus stock was extracted, DNase treated, and reverse transcribed, and long-range PCR was performed using primers specific to the first 25 nt of the 3′-end leader sequence and the last 30 nt of the 5′ trailer region (data not shown). This approach would allow amplification of genomes that contain a large internal deletion(s). No short amplicons were detected from wt rRSV RNA, but short amplicons of various sizes and amounts were detected from each of the four Max viruses as well as from rRSV/Δ1313/I1314L (data not shown). Each virus generated a unique pattern, with no evident amplicons common between viruses. Eleven individual amplicons (with the following numbers per virus: Max A, 1; Max B, 3, Max L, 1; Max FLC, 3; and rRSV/Δ1313/I1314L, 3) were purified on agarose gels and subjected to sequence analysis with each of the two primers used to generate the amplicons. In most cases, specific sequences were not detected above background. Only three amplicons yielded clean sequences: two from Max B and one from Max L. One of the Max B amplicons yielded sequence that was identified as being from the pBluescript vector, whereas the other two amplicons contained RSV sequence. The Max B amplicon was 822 nt long and contained nt 1 to 49 fused to nt 14451 to 15223 (numbered with nt 1 being the first leader nucleotide and nt 15223 being the last trailer nucleotide). The Max L amplicon was 1,065 nt long and contained nt 1 to 227 fused to nt 14386 to 15223. These data suggest that two of the Max virus stocks, Max B and Max L, might contained viral genomes with large internal deletions.

## DISCUSSION

Previous studies of RNA viruses in which codon pair usage was modified usually involved deoptimization, namely, increasing the content of codon pairs that are underrepresented in the human ORFeome. Deoptimization typically results in virus attenuation. A major factor in this attenuation has been thought to be reduced efficiency of translation (6, 20, 21). More recently, it has been suggested that part of the attenuation from CPD may be due to an increased content of CpG and UpA residues capable of stimulating host immune responses (3, 22–25). In the case of RSV, the effects of CPD appear to include direct impairment of the virus (11). Specifically, each of four CPD RSV mutants that were made with different CPD ORFs were found to be ts. Following selective passage in cell culture, a variety of mutations in various viral proteins that substantially reversed the ts phenotype and paradoxically increased the *in vivo* attenuation phenotype were identified. These observations are more compatible with direct effects of CPD on viral rather than host factors. Of note, there also could be an additional contribution to attenuation from CpG and UpA dinucleotides. With these precedents in mind, in the present study, we investigated the effect of CPO on RSV biology.

Because of the formal possibility that CPO might result in increased RSV virulence, due to the expected increased viral protein synthesis, these studies were performed in a viral backbone that was strongly attenuated by a genetically stabilized attenuating ts mutation in the L polymerase. In addition, due to prior RSV infection, essentially all humans worldwide over the age of 2 to 3 are seropositive for RSV, which provides an additional safety factor.

Compared to the isogenic parent virus rRSV/Δ1313/I1314L, we found that CPO of RSV ORFs had minimal or no effect on the following virus characteristics: the kinetics and efficiency of virus replication *in vitro*, ts phenotype (except for a 1°C downward shift for Max FLC), and specific infectivity (the ratio of PFU to genomes). This absence of effect is in contrast to our previous study of CPD of RSV ORFs, in which each of the four CPD viruses exhibited a restricted replication *in vitro* and a ts phenotype and the Min B and Min FLC viruses also exhibited 13,900- and 24-fold increases in the ratio of genomes per PFU, respectively, compare to the wt, suggestive of an increase in noninfectious particles (11). The algorithm that was used to recode RSV ORFs rearranges the existing codons to generate overrepresented codon pairs without affecting the folding free energy of the RNA, and thus no changes in viral RNA architecture would be expected following CPO. Indeed, all CPO viruses replicated similarly to rRSV/Δ1313/I1314L (the parallel non-CPO control) in multicycle replication experiments, suggesting that no gross effects on viral RNA or nucleocapsid architecture occurred due to CPO.

The Max A and Max L viruses appeared to have a modest increase in the synthesis of positive-sense RNAs at 16 hpi, although by 24 hpi the differences had disappeared. Max A and Max L also exhibited a global increase in viral protein expression. The CPO ORFs in these viruses included those for N and P in the case of Max A and L in the case of Max L. The proteins N, P, and L are part of the RSV polymerase complex. It is tempting to speculate that CPO of these ORFs resulted in a modest increase in the synthesis of the RSV replication complex, leading to a modest increase in global viral transcription during most of the single-cycle replication period, which in turn increased global viral protein expression. The idea that CPO can increase protein synthesis is consistent with previously published data suggesting that changes in CPB affect protein translation (2). More specifically, the use of overrepresented codon pairs increases protein expression (6).

However, in our study, CPO did not always increase the efficiency of gene expression. In particular, CPO of the G and/or F ORF substantially reduced global viral transcription, viral protein synthesis, and single-cycle virus replication. We previously showed that CPD of F and G strongly reduced virus replication and the specific infectivity of RSV (11). Thus, in aggregate, our CPD and CPO data suggest that RSV is intolerant of either decreases or increases in the CPB of the G and F ORFs.

The formal possibility existed that CPO of the G and/or F ORF to make Max B had inadvertently introduced a sequence that somehow was highly inhibitory to transcription, translation, RNA replication, or RNA stability. This inhibition hypothesis was investigated by constructing minigenomes that individually expressed the wt or CPO G or F ORF. Unlike the situation for complete Max B virus, in this minigenome system, RNA replication and gene transcription are driven by helper plasmids and are independent of expression of the G or F ORF contained in the minigenome. Therefore, the efficiencies of expression of the wt and CPO G and F ORFs could be directly compared. The comparison did not detect any effects of CPO on glycoprotein expression, suggesting that the effects of CPO of the RSV G and F ORFs were not due to the inadvertent introduction of an artifactual inhibitory sequence. It is noteworthy that the algorithm used in the CPO avoided changing the RNA folding free energy, and therefore major changes in RNA folding, which might affect expression, were avoided. One change that did occur is that a number of homopolymer stretches (of 6 to 7 nt in length), which are characteristic of the RSV genome, were interrupted by CPO. For example, in the case of G and F, CPO removed 3 U homopolymers in G and 1 A homopolymer in F. It is formally possible that these changes somehow affected Max B gene expression and/or protein synthesis, resulting in the reduced virus replication. To investigate this, these stretches would have to be reintroduced back individually and in combination into the Max B backbone and their effect on virus replication evaluated.

Surprisingly, replication of CPO RSVs was slightly reduced in rodents. Even though the differences were not significant statistically, there was a consistent trend in the data. These data showed that the use of overrepresented codon pairs did not improve viral replication and that the wt virus appeared to be slightly fitter for replication than the CPO viruses. More surprisingly, we found that the antibody response was reduced in hamsters infected with CPO rRSV compared with wt rRSV-infected hamsters. The reduction in the antibody response was greater with increased numbers of CPO ORFs. The magnitude of the reduction in antibody titer was greater than the reduction in viral replication. The former was statistically significant, while the latter was not. Thus, while it is possible that the reduction in immunogenicity was due to the trend toward reduced replication, resulting in reduced antigen load, these data suggest that the CPO of RSV influenced the antibody response.

In addition, we observed that the level of replication and protein expression by the CPO viruses *in vitro* sometimes was inconsistent with their replication and immunogenicity *in vivo*. As discussed above, Max B replication was reduced compared to that of other CPO viruses in single-cycle replication experiments *in vitro*, and Max B also had reduced G and F expression. However, this virus replicated similarly to other CPO viruses in hamsters and was substantially immunogenic (i.e., equivalent to that of Max L). As another example, Max A replicated efficiently *in vitro* and was more efficient in protein expression than wt rRSV but had reduced replication and immunogenicity *in vivo*. In a previous study in which we subjected RSV to codon pair deoptimization (CPD) (26), we also observed inconsistencies between the efficiency of replication and protein expression of some CPD RSVs *in vitro* and their replication and immunogenicity *in vivo*. For example, RSV that contained a CPD L ORF with three additional stabilizing mutations replicated less efficiently than wt rRSV *in vitro* and *in vivo* and yet was similar to wt rRSV in immunogenicity *in vivo*. These observations indicate that RSV viruses with changes in their codon pair bias (CPO or CPD) can exhibit unexpected differences between replication and protein expression *in vitro* versus replication and immunogenicity *in vivo* that remain to be understood.

We also performed a preliminary analysis to detect possible defective genomes in these virus stocks, which might interfere with virus replication (18) but also might stimulate the innate and adaptative immune responses (19, 27, 28). Short amplicons were detected in rRSV/Δ1313/I1314L, Max A, Max B, Max, L and Max FLC stocks but not for wt RSV. However, only two amplicons (detected in stocks of Max B and Max L) contained RSV sequence. The presence of defective genomes in any of these virus stocks was surprising because the stocks were low-passage: specifically, the transfected BSR T7/5 cells were cocultured with Vero cells, followed by a single passage in Vero cells (see Materials and Methods). Interestingly, the sequencing of the two short RSV-specific amplicons mapped the presumptive downstream break points to the 5′ end of the full-length genome (between nt 14386 and 14451 in the L ORF) in a region that was previously shown to favor the generation of truncated genomes (29). Additional experiments would be needed to confirm that these short amplicons are indeed defective genomes and not PCR artifacts due to primers mismatching, to investigate whether the other type of defective genome (copyback) can be detected, to evaluate the effect of these defective genomes on viral replication (possibly causing a decrease) and immunogenicity (possibly causing an increase), and to determine whether enhanced production of defective genomes is linked to CPO or the presence of the Δ1313/I1314L attenuating mutations in the L polymerase.

A plausible explanation would be that CPO rRSVs with reduced frequencies of CpG and UpA dinucleotides (about 20% and 16% reductions in Max FLC, respectively, compared to the wt) would, directly or indirectly, less efficiently stimulate the activation and proliferation of B cells. Indeed, it is well known that CpG DNA can directly and very efficiently stimulate B cells but also natural killer cells, dendritic cells (DC), and monocytes/macrophages through Toll-like receptor (TLR) stimulation (16). In addition, synthetic CpG RNAs have been shown to stimulate human monocytes, resulting in interleukin-6 (IL-6) and IL-12 production and costimulatory molecule upregulation (17). During virus transcription, large amounts of viral mRNAs or double-stranded intermediates that could be potentially recognized by the innate immune response are produced. Double-stranded RNA (dsRNA) is recognized through TLR3 and is a potent stimulator of human monocytes and myeloid DC, while single-stranded RNAs (ssRNAs) were shown to activate myeloid DC.

Consistent with the importance of CpG RNAs, while CPO rRSVs poorly induced an antibody response in hamsters, CPD rRSVs, which contained an increased number of CpGs and UpAs compared with those in wt rRSV, induced *in vivo* an antibody response that was equivalent to or even higher than that of the wt despite a strong reduction of replication (11, 26). Such an increase in immunogenicity by CPD has also been described in other virus models (21, 30), suggesting that the CPB of a virus may affect the antibody response of the host, possibly through an increase of the CpG and UpA content. In addition, this effect on the antibody response could explain why virus genomes exhibit low CpG and UpA contents. All together, our data suggest that manipulation of the CPB of an RNA virus could ultimately be used as a tool to generate RNA viruses with modified immunogenicity.

## MATERIALS AND METHODS

### Cells.

African green monkey kidney Vero cells were grown in Opti-MEM I as described previously (11). BSR T7/5 cells, a derivative of baby hamster kidney 21 (BHK-21) cells that constitutively expresses T7 RNA polymerase (31), were grown in Glasgow minimal essential medium (GMEM) (Gibco-Life Technologies) as described previously (11). Human airway epithelial A549 cells were grown in F12 medium (HyClone) with 10% fetal bovine serum (FBS) and 1% l-glutamine. Cells were maintained at 37°C with 5% CO_2_.

### Recoding the genes of RSV.

Using a computer algorithm (6), the RSV ORFs were computationally recoded to contain a large number of codon pairs that are overrepresented in the human ORFeome without altering the overall codon usage, the predicted free energy of RNA folding, or the amino acid sequence of RSV. The process of CPO introduced a few nucleotide homopolymers of >6 nt as well as sequences similar to the conserved RSV gene start and gene end signals, and these were manually removed. In addition, the first 30 bases of each ORF were kept identical to those of wild-type (wt) RSV in order to avoid effects on initiation of transcription or translation. Four CPO genomes were constructed (Fig. 1A): Max A, with CPO of the NS1, NS2, N, P, M, and SH ORFs; Max B, with CPO of the G and F ORFs; Max L, with CPO of the L ORF; and Max FLC, in which all of the ORFs were CPO except for M2-1 and the overlapping M2-2 ORF. For safety against the possibility of increased viral fitness, each CPO genome was attenuated by the addition of the codon deletion mutation Δ1313 and the missense mutation I1314L in the L polymerase gene (see below). RSV genome segments bearing CPO ORFs were synthesized commercially. This project was reviewed and approved by the NIH Deputy Director for Intramural Research’s Dual Use Committee.

### Construction of cDNAs encoding CPO recombinant RSVs.

Recombinant RSVs bearing CPO ORFs were constructed based on the antigenome cDNA D46/6120, a derivative of the strain A2 antigenomic cDNA (GenBank accession number KT992094) with the deletion of a 112-nt fragment of the 3′ noncoding region of the SH gene and five synonymous codon changes in the last three codons and the stop codon of the SH ORF (32). These changes were made to improve cDNA stability during amplification of RSV full-length plasmids in Escherichia coli. Virus replication *in vitro* or in mice was not significantly altered by these changes (32). For simplicity, the numbering of sequence positions in the present article is based on the complete sequence of biologically derived strain A2 (GenBank accession number M74568). Max A was constructed by replacing a 4,508-bp NotI-XhoI fragment of D46/6120 with a synthetic cDNA containing the CPO genes (Fig. 1A). Construction of Max B involved replacement of a 3,907-bp XhoI-BamHI cDNA fragment, and that of Max L involved replacement of a 6,750-bp BamHI-KasI fragment. Finally, Max FLC was generated by successively transferring all three synthetized CPO fragments (NotI-XhoI, XhoI-BamHI and BamHI-KasI) into D46/6120. The Δ1313/I1314L mutations (13) were introduced into the L gene of the wt, Max A, Max B, Max L, and Max FLC cDNA backbones using the QuikChange Lightning site-directed mutagenesis kit (Agilent) following the manufacturer’s recommendations. cDNAs were completely sequenced by automated Sanger sequencing using a set of specific primers.

### Virus recovery, growth, and titration.

CPO viruses were rescued from cDNA as described previously (11). Briefly, BSR T7/5 cells were transfected at 37°C using Lipofectamine 2000 (Thermo Fisher) and a plasmid mixture containing 5 μg of full-length cDNA, 2 μg each of pTM1-N and pTM1-P, and 1 μg each of pTM1-M2-1 and pTM1-L (33, 34). After overnight incubation at 37°C, transfected cells were harvested by scraping into medium, added to subconfluent monolayers of Vero cells, and incubated at 32°C. The rescued viruses were harvested between 11 and 14 days posttransfection, when the monolayer began to deteriorate visibly. The viruses were then propagated by a single passage on Vero cells at 32°C in medium containing 2% FBS. The monolayers were incubated until viral cytopathic effect was evident, at approximately 5 to 7 days postinfection (p.i.). Virus stocks were generated by scraping infected cells into medium, followed by vortexing for 30 s to release surface-associated virus, followed by clarification of the supernatant by centrifugation. Virus aliquots were snap-frozen and stored at −80°C. Virus titers were determined by plaque assay on Vero cells with an 0.8% methylcellulose overlay followed by immunostaining with a mixture of three RSV-specific monoclonal antibodies (MAbs) (35). Titers were expressed as PFU per ml. Viral RNA was isolated from all virus stocks, and automated sequence analysis of the viral genomes was performed from overlapping uncloned RT-PCR fragments, confirming that the genomic sequences of the recombinant viruses were correct and free of adventitious mutations. The only sequences that were not directly confirmed for each genome were the positions of the outermost primers, namely, nucleotides 1 to 23 and 15174 to 15222.

### Multicycle replication *in vitro*.

Multicycle virus replication experiments were performed in Vero and A549 cells (in 6-well and 12-well plates, respectively). Wells were infected in duplicate at a multiplicity of infection (MOI) of 0.01 PFU/cell and incubated at 32 or 37°C. Viruses were harvested daily from day 1 to 10 (Vero cells) or from day 1 to 7 (A549 cells) by scraping infected cells into medium, followed by vortexing for 30 c and clarification by low-speed centrifugation. Two wells were processed individually for each sample/time point. Virus aliquots were snap-frozen and stored at −80°C until titers were determined, as described above, in parallel with samples of the inocula to confirm titers.

### Single-cycle replication *in vitro*.

A single-cycle virus replication experiment to evaluate viral RNA synthesis, protein synthesis, and virus production in parallel was performed as follows. Replicate cultures of Vero cells in 6-well dishes were mock infected or infected at an MOI of 3 PFU/cell with the indicated viruses and incubated at 32°C or 37°C as indicated. Cultures were washed once after the first 2 h of incubation. Wells were harvested at 4, 10, 16, 24, and 36 hpi for the following parallel analyses: (i) a single well for each virus/time point was harvested, from cells incubated at 32°C or 37°C, for analysis by RT-qPCR (see below); (ii) a single well for each virus/time point was harvested, from cells incubated at 32°C, for analysis by flow cytometry (see below); and (iii) a single well for each virus/time point was harvested, from cells incubated at 32°C or 37°C, by scraping and clarification as described above and processed for quantification of infectious virus by plaque assay as described above. At 24 h a single well for each virus was harvested, from cells incubated at 32°C, for Western blot analysis (see below), and two additional single-cycle replication experiments were performed to obtain two repetitions of the Western blot and virus production assays. In each of these two additional experiments, Vero cells were infected with an MOI of 3 PFU/cell or mock infected at 32°C, as described above. At 24 hpi, a single well for each virus was harvested for Western blot analysis and a single well for each virus was harvested and processed for virus titration.

### Reverse transcription and strand-specific qPCR.

Cell monolayers from the single-cycle experiment described above were harvested to isolate total cell-associated RNA using the RNeasy minikit (Qiagen), treated with DNase I to remove residual genomic DNA, and analyzed by reverse transcription and PCR to quantify viral negative-sense (genomic) and positive-sense (mRNA and antigenomic) RNA. The M2 gene was used as a common target because it was the only gene that remained unchanged in the wt and CPO rRSVs. In addition, the expression of the P, G, F, and L genes by CPO or wt viruses was also investigated with a specific set of primers for wt or CPO ORFs. Primers and probes were designed using Primer 3 software (Thermo Fisher) and are available upon request. Strand-specific quantitative TaqMan-based RT-PCR (RT-qPCR) assays were performed using tagged primers as described previously (11, 26). qPCR results were analyzed using the comparative threshold cycle (Δ*C_T_*) method, normalized to 18S rRNA, and then expressed as fold increase over that for rRSV/Δ1313/I1314L at the 4-h time point at 32°C for wt genes or expressed as fold increase over that for Max FLC at the 4-h time point at 32°C for CPO genes.

### Analysis of virus proteins expression by flow cytometry.

Cell monolayers from the single-cycle replication experiments were harvested by trypsin treatment, washed in phosphate-buffered saline (PBS), and stained with a far-red live/dead dye (Thermo Fisher) before being fixed using the Cytofix/Cytoperm fixation buffer (BD Biosciences). Cells were then resuspended in perm/wash buffer (BD Biosciences) and stained with a mixture of the following antibodies for analysis of the intracellular RSV protein expression: a fluorescein isothiocyanate (FITC)-labeled anti-RSV P MAb (Abcam), an allophycocyanin (APC)-labeled anti-RSV N MAb (Imgenex), a Brilliant Violet 421 (BV421)-labeled anti-RSV G MAb (Abcam), and a biotin-labeled anti-RSV F MAb (Millipore). Staining was performed for 1 h at 4°C in the dark with MAbs at concentrations previously determined to be optimal by titration. After incubation, cells were extensively washed with perm/wash buffer, stained with a pretitrated concentration of streptavidin-BV605, and then incubated in the dark for 30 min at 4°C. After incubation, cells were extensively washed in perm/wash buffer and resuspended in 300 μl of PBS for flow cytometry analysis. Thirty thousand live single cells were acquired using a BD Fortessa flow cytometer (BD Biosciences). Compensation was performed automatically using single-color-labeled cells for each antibody. The gating was performed generously (i.e., far enough from the negative populations to not include negative events in the positive gates). Live/dead staining, forward scatter height, and forward scatter area were used to identify single live cells, and then the expression of virus proteins N, P, G, and F was analyzed on single live cells using FlowJo version 15 software (Tree Star, Inc., Ashland, OR).

### Western blot analysis.

Cell monolayers from the single-cycle experiment described above were lysed using a nondenaturing and nonreducing cell lysis buffer containing 1× protease inhibitor (Roche) and homogenized using a QIAshredder (Qiagen). Clarified cell lysates were adjusted to contain 1× NuPAGE reducing agent (Thermo Fisher) and denatured by heating for 10 min at 90°C. The samples were separated on NuPAGE 4 to 12% Bis-Tris SDS-polyacrylamide gels with morpholineethanesulfonic acid (MES) electrophoresis buffer (Life Technologies) in parallel with the Odyssey two-color protein molecular weight marker (Li-Cor). Proteins were transferred to polyvinylidene difluoride (PVDF) membranes (Millipore). The membranes were blocked with Odyssey blocking buffer (Li-Cor) and incubated overnight at 4°C with primary antibody in the presence of 0.1% Tween 20. The primary N, P, G, and F monoclonal antibodies (Abcam) were used at a 1:1,000 dilution. Rabbit anti-glyceraldehyde-3-phosphate dehydrogenase (anti-GAPDH) polyclonal antibody (Santa Cruz Biotechnologies, Inc.) was used as loading control at a 1:200 dilution. The secondary antibodies, used at a 1:15,000 dilution, were goat anti-rabbit IgG IRDye 680 (Li-Cor) and goat anti-mouse IgG IRDye 800 (Li-Cor). Membranes were scanned on the Odyssey infrared imaging system. Data were analyzed using Odyssey software, version 5.2.5 (Li-Cor). For quantification of identified RSV proteins of interest, fluorescence signals were corrected for background. Values used are the background-corrected fluorescence intensity (FI) of each protein band.

### Evaluation of the replication of CPO rRSVs in mice.

All animal studies were approved by the National Institutes of Health (NIH) Institutional Animal Care and Use Committee (ACUC). Using previously described methods (11), groups of 10 mice per virus (or 5 for wt rRSV) were inoculated intranasally (i.n.) under isoflurane anesthesia with 10^6^ PFU of the indicated virus. On days 4 and 5 (day 5 only for wt rRSV), five mice from the indicated group were sacrificed by carbon dioxide inhalation. Nasal turbinates (NT) and lung tissues were harvested and homogenized separately in Leibovitz 15 (L-15) medium containing 1% l-glutamine, 0.06 mg/ml clindamycin phosphate, 0.05 mg/ml gentamicin, and 0.0025 mg/ml amphotericin B. Virus titers were determined in duplicate on Vero cells incubated at 32°C as described above. The limits of virus detection were 100 and 50 PFU/g for the NT and lung specimens, respectively.

### Evaluation of the replication and immunogenicity of CPO rRSVs in hamsters.

Six-week-old golden Syrian hamsters in groups of 12 were inoculated i.n. under methoxyflurane anesthesia with 10^6^ PFU of the indicated viruses. On day 3, which corresponds to the peak of replication of wt RSV in hamsters (26), 6 hamsters from each group were sacrificed by carbon dioxide inhalation. NT and lung tissue were harvested and homogenized as described above. Virus titers were determined in duplicate on Vero cells incubated at 32°C as described above. The limit of virus detection was 50 PFU/g in the NT and lungs. On day 25 p.i., sera were collected from the remaining six hamsters per group. On day 29, the hamsters were challenged by i.n. administration of 10^6^ PFU of wt rRSV. Three days after challenge, the hamsters were sacrificed by carbon dioxide inhalation. NT and lung tissue were harvested, and challenge wt rRSV titers were determined in duplicate on Vero cells incubated at 37°C as described above.

### Statistical analysis.

Data sets were assessed for significance using the nonparametric Mann-Whitney or Kruskal-Wallis test with Dunn’s *post hoc* test. Statistical analyses were performed using Prism 5 (GraphPad Software). Data were considered significant only at a *P* value of ≤0.05.

### Data availability.

The GenBank accession numbers of the Max A, Max B, Max L, and Max FLC full-length nucleotide sequences are MK733766, MK733767, MK733768, and MK733769, respectively. Note that the Max A, Max B, Max L, and Max FLC full-length nucleotide sequences do not contain the Δ1313/I1314L mutations.
